# Endometriosis Associated Infertility: A Critical Review and Analysis on Etiopathogenesis and Therapeutic Approaches

**DOI:** 10.3390/medicina56090460

**Published:** 2020-09-09

**Authors:** Lidia Filip, Florentina Duică, Alina Prădatu, Dragoș Crețoiu, Nicolae Suciu, Sanda Maria Crețoiu, Dragoș-Valentin Predescu, Valentin Nicolae Varlas, Silviu-Cristian Voinea

**Affiliations:** 1 Alessandrescu-Rusescu National Institute for Mother and Child Health, Fetal Medicine Excellence Research Center, 020395 Bucharest, Romania; lidia.filipsd@gmail.com (L.F.); flory_duica@yahoo.com (F.D.); neagualina0206@yahoo.ro (A.P.); dragos@cretoiu.ro (D.C.); nsuciu54@yahoo.com (N.S.); 2 Department of Cell and Molecular Biology and Histology, “Carol Davila” University of Medicine and Pharmacy, 050474 Bucharest, Romania; sanda@cretoiu.ro; 3 Department of Obstetrics and Gynecology, “Carol Davila” University of Medicine and Pharmacy, 05047 Bucharest, Romania; 4 Department of Obstetrics and Gynecology, Alessandrescu-Rusescu National Institute for Mother and Child Health, Polizu Clinical Hospital, 020395 Bucharest, Romania; 5 Department of General Surgery, Sf. Maria Clinical Hospital, “Carol Davila” University of Medicine and Pharmacy, 011172 Bucharest, Romania; 6 Department of Obstetrics and Gynecology, Filantropia Clinical Hospital, 01171 Bucharest, Romania; 7 Faculty of Dental Medicine, “Carol Davila” University of Medicine and Pharmacy, 030167 Bucharest, Romania; 8 Department of Surgical Oncology, Alexandru Trestioreanu Oncology Institute, “Carol Davila” University of Medicine and Pharmacy, 022328 Bucharest, Romania; dr.voineasilviu@gmail.com

**Keywords:** endometriosis, infertility, etiopathogenesis, treatment, in vitro fertilization

## Abstract

Endometriosis represents a frequently diagnosed gynecological affliction in the reproductive timespan of women, defined by symptoms ranging from pelvic pain to infertility. A complex interplay between the genetic profile, hormonal activity, menstrual cyclicity, inflammation status, and immunological factors define the phenotypic presentation of endometriosis. To date, imaging techniques represent the gold standard in diagnosing endometriosis, of which transvaginal ultrasonography and magnetic resonance imaging bring the most value to the diagnostic step. Current medical treatment options for endometriosis-associated infertility focus on either stimulating the follicular development and ovulation or on inhibiting the growth and development of endometriotic lesions. Techniques of assisted reproduction consisting of superovulation with in vitro fertilization or intrauterine insemination represent effective treatment alternatives that improve fertility in patients suffering from endometriosis. Emerging therapies such as the usage of antioxidant molecules and stem cells still need future research to prove the therapeutic efficacy in this pathology.

## 1. Introduction

Endometriosis is a challenging condition of reproductive-aged women, causing problems ranging from chronic pain to infertility. It is characterized by an estrogen-dependent stroma and endometrial glands found predominantly, but not exclusively, in the pelvic compartment [1]. Due to the necessity of surgical visualization for a definite and clear diagnostic, a precise evaluation of the prevalence and incidence of the disease is hard to obtain [2]. This disease is characterized by a prevalence estimated at 5%, peaking between 25 and 35 years [3], and an annual incidence among women aged 15–49 years, evaluated at 0.1% [4], generating thus significant healthcare costs (according to a study conducted by Simoens et al., the average annual cost per woman was estimated at €9579 [5]).

Although the relation between endometriosis and infertility as a matter of a definitive cause-effect connection is still debatable, it is clinically recognized and well supported throughout the literature [6]. Currently, endometriosis-associated infertility is viewed as a multifactorial problem, facing matters related to altered immunity and genetics, that affects not only the fallopian tubes and the embryo transport but also the normal endometrium [7]. To date, treating infertility caused by endometriosis is focused on removing or reducing ectopic endometrial implants and restoring the normal pelvic anatomy either by medical, surgical, or assisted reproductive technologic means [8]. The medical approach targets the ovarian function, blocking it with various drugs such as agonists of gonadotropin-releasing hormone and oral contraceptives [9]. Assisted reproductive technologies (ART), such as in-vitro fertilization (IVF) come into play when neither medical nor surgical attempts meet the required outcome [10]. IVF has been demonstrated to represent one of the key treatment options for patients suffering from endometriosis-associated infertility, especially when it involves a compromised tubal function, aberrant peritoneal anatomy, or failure of other treatment methods [11].

This manuscript constitutes a critical review and analysis of the literature and focuses on the epidemiological and clinical aspects of endometriosis as a disease and the current tools used for diagnosing this ailment, as well as several proposed mechanisms for infertility development and its current treatment options.

## 2. Endometriosis—Epidemiological and Clinical Overview

### 2.1. Risk Factors for Developing Endometriosis

Even though significant insight was gained on the disease through research initiatives in the last decades, the exact cause of endometriosis remains unclear. It has been accepted that the genetic profile, hormonal activity, inflammation status, and immunological environment, play an important role in the manifestation and progression of endometriosis [12]. From an epidemiological perspective, it is regarded currently that an intertwined relationship between socio-economic status, family history, constitutional factors, personal habits, reproductive and gynecological status, as well as environmental factors, constitutes one of the first steps regarding the occurrence of endometriosis [2,13].

Concerning the environmental factors, it has been suggested that exposure to elevated levels of polychlorinated biphenyls [14], dioxin [15], phthalate esters, bisphenol A [16] or organochlorinated pollutants and perfluorochemicals may play a role in the development of endometriosis. Some theories regarding the mechanism of action by which these pollutants are involved in initiating the disease include generating oxidative stress which can modulate the immunological activity or alter the hormonal homeostasis, however, further research is needed to shed light on the exact pathways of the intervention of these toxins regarding endometriosis [13].

As for behavioral traits, the relationship between dietary preferences, alcohol and caffeine intake, smoking, and physical activity in regards to involvement in developing endometriosis has been studied [17]. Some dietary preferences, generally related to consumption of red meat, have been associated with higher incidence related to developing endometriosis, while others, such as consumption of fresh fruits and vegetables, have shown to diminish this risk [18]. It has been hypothesized that caffeine and alcohol intake might play a role in endometriosis pathogenesis by altering reproductive hormones via aromatase activation which increases the conversion of testosterone to estrogen [19,20]. While it is well known that smoking increases the inflammatory status and alters the hormonal balance, one meta-analysis concluded that there is little evidence to link tobacco smoking to endometriosis, even in the case of heavy users [20]. Taking into account the risk of physical activity on endometriosis, it has been hypothesized that intense physical activities might stimulate endometrial proliferation by increasing estrogen levels and insulin-like growth factor-1 [21], while normal intensity exercise might have a protective effect by reducing the inflammatory status and oxidative stress [22].

In terms of reproductive and gynecological factors, most of the risk factors associated with endometriosis focus on age at menarche, menstrual cycle length, duration of flow, and parity [23]. Menarche starting at an early age and long and heavy menstrual cycles have been correlated with higher risk due to higher concentrations of estradiol and estrone [24], while parity and oral contraceptive usage [25] were related to protective status. Even though tubal ligation has been thought to decrease the risk of the disease by inhibiting endometrial cells to reach the pelvic compartment [26], patients receiving this treatment might represent a biased group of asymptomatic patients with fewer gynecologic and reproductive problems than women seeking nonsurgical methods of contraception, making the association difficult to interpret [18]. 

Global efforts combining genetic and genomic technologies have been made to provide insight into the pathophysiology of this disease, resulting in the discovery of several regions linked to an increased risk of endometriosis [27]. To date, research teams have identified 14–16 genomic regions correlated with a higher risk in the occurrence of endometriosis. It has been acknowledged that chromosomes 1, 9, 12, display several potential target genes such as LINC00339, CDC42, CDKN2A-AS1, and VEZT, having altered gene regulation [28]. Nevertheless, future studies are necessary to identify target genes in each region to determine how alterations of these targets work to increase the risk of developing endometriosis.

Furthermore, recent insights have been gained through the study of this disease from a genetic/epigenetic perspective, suggesting that the endometriotic lesions might develop by implantation of floating endometrial cells possessing inherited and non-expressed DNA defects, a process aided by other factors such as immunologic imbalances, inflammation, and oxidative stress disturbances [29].

### 2.2. Clinical Features of Endometriosis

The clinical presentation of reproductive-aged women suffering from endometriosis is highly variable, with symptoms ranging from pelvic pain, dysmenorrhea, to infertility [30], thus having a serious impact on the mental and socioemotional well-being of the patient [1]. Pelvic pain is characterized by a dull, sharp, throbbing, or burning sensation, while dysmenorrhea is described as pelvic pain that presents before, during, and/or after menstruation. Compression and infiltration of the nerves in the endometrial lesions represent the main cause of pain development in endometriosis, although other mechanisms such as the nociceptive pain component of neuropathic pain, neurogenic inflammatory processes, and myogenic pain along with alterations in peripheral and central nervous system pain processing have been considered in generating the endometriosis-associated pelvic pain complex pathology [31,32]. One research team from Brazil proposed a theory of perineural spread in 2015, in which they concluded that several interactions occur at the subcellular level between endometriosis lesions and somatic peripheral nerves [33]. Regarding the correlation between pain and the severity of the disease, new studies suggest that minor or mild stages of the disease are mostly associated with pain in form of dysmenorrhea, dyspareunia, pelvic pain, back pain, or fatigue, while in more advanced stages of impairment, painful defecation, micturition, and blood in the urine add up to the clinical presentation [34].

Strenuous efforts have been made in order to present a clear classification of the disease, nevertheless, the World Endometriosis Consensus decided in 2014 that the best classification model should be a toolbox consisting of several other classification systems including the American Society for Reproductive Medicine (rASRM) and the Enzian classifications along with the endometriosis fertility index (EFI) [35]. rASRM is designed for women suffering from endometriosis concerned for their fertility status and takes into consideration the level of ovarian and peritoneal involvement as well as posterior cul-de-sac obliteration, with scores ranging from 1 to 5 for minimal disease, 6–15 for mild impairment, 16–40 corresponding to moderate, and over 40 severe disease, respectively [35]. The Enzian classification focuses on staging the deep infiltrating lesions taking into consideration the cul-de-sac, vagina, uterosacral ligament, cardinal ligament, bowel, and the rectosigmoid areas [36]. EFI has been developed in hopes of improving the estimation of the fertility status and it uses a combination of laparoscopic data with anamnesis details such as age, prior pregnancies, and the number of years while suffering from infertility [37]. Nevertheless, this toolbox classification has poor predictive potential in regards to prognosis of the disease as well as with the clinical presentation, future optimizations being needed to perfect this diagnostic algorithm [35].

The constellation of symptoms relays mostly on the anatomic sites of involvement, represented in decreasing order of frequency by the ovaries, anterior and posterior cul-de-sac, posterior broad ligaments, uterosacral ligaments, uterus, fallopian tubes, sigmoid colon and appendix, and round ligaments [38]. Endometriosis can occur in less common sites such as umbilicus [39], gastrointestinal structures like the cecum, and the ileum or genitourinary elements such as the urinary bladder, ureters, vagina, cervix, or the rectovaginal septum [40]. The rarest areas of anatomical distribution have been reported on the hymen [41], the lung [42], nerves located in the pelvic compartments, as well as on surgical scars [43]. Based on the location of the endometrial implants, it is generally acknowledged that this disease can be divided into three categories such as peritoneal endometriosis, ovarian endometriosis, and deeply infiltrated endometriosis, each one this different pathogenesis presenting different invasive and proliferative mechanisms [44].

As for the clinical presentation of endometriosis located outside the gynecological sphere, bladder involvement can cause recurrent dysuria and suprapubic pain, and cyclic microscopic hematuria when localized in the ureters [45,46]. Gastrointestinal endometriosis can manifest as cyclical abdominal pain, meteorism, tenesmus, constipation, melena, diarrhea, vomiting, and hematochezia [47] but also as dyspareunia and painful defecation when lesions are localized in the posterior cul-de-sac and rectovaginal septum [48]. When found in unusual anatomical sites such as the thoracic cavity, endometriosis manifests as chest pain, hemoptysis or pneumothorax, or hemothorax [49,50]. Therefore, due to the complexity of symptoms, endometriosis can mimic a wide number of variate diseases and it is necessary that thorough anamnesis be coupled with other specific diagnostic tools for an exact diagnostic.

### 2.3. Diagnostic Tools for Endometriosis

Diagnosing endometriosis requires a vast array of tools related not only to clinical assessment but also to biological clues as well as imaging techniques, whether non-invasive such as ultrasonography or surgical methods for direct visualization. Evaluating the presence of symptoms and performing a physical examination represent the first steps in diagnosing endometriosis [51,52]. As mentioned, before, symptoms of endometriosis correlate with the site of anatomical involvement, thus, women are complaining most frequently of cyclic pelvic pain in the form of dysmenorrhea, intermenstrual pain, and dyspareunia.

Physical examination, while executing the bimanual pelvic maneuver, points toward diagnosing endometriosis when several criteria are met such as palpable nodularity and abnormal pelvic anatomy, especially when located in the vagina and the rectovaginal space, the pouch of Douglas, the rectosigmoid, as well as the posterior wall of the urinary bladder [44,51,52]. Other signs such as tenderness, decreased mobility, and a retroverted uterus, evidenced while palpating, might also indicate signs of endometriosis [53]. During speculum inspection, endometriosis might be present in form of red or blue hypertrophic and hemorrhagic nodules, usually in the posterior fornix [54]. Nevertheless, a normal clinical examination does not eliminate the diagnosis of endometriosis, further investigations such as laboratory and imaging techniques being required to assess the extent and impact of the disease.

In regards to laboratory findings, strenuous research has been made to identify the serum markers which can be used as screening tests for endometriosis, yet currently, none of the discoveries can be accounted as an effective tool due to inadequate sensitivity and specificity indexes [55]. To date, several potential biomarkers and their relation to endometriosis have been investigated, including inflammatory cytokines [56], growth factors [57], angiogenesis markers [58], stem cell markers [58], steroids and hormones [59], tissue matrix metalloproteinases and adhesion molecules [60,61], nevertheless, none of them proved to be a reliable diagnostic tool.

A special interest has been shown towards CA-125, a high-molecular-weight glycoprotein antigen expressed in some derivatives of the celomic epithelium [62], used as a marker for epithelial cell ovarian cancer [63]. It has been reported that CA-125 was present in elevated serum levels in patients with advanced forms of endometriosis [64,65]. Numerous studies have been conducted to prove the sustainability of using this biological marker for endometriosis, due to its good specificity (93%) at values ≥ 30 units/mL [65], even though the sensitivity showed to be low (53%) [66].

Imaging techniques have an important role in aiding the endometriosis diagnostic, whether non-invasive or invasive in nature, due to their power of pointing the exact location of lesions and assessing the disease extent. In this regard, numerous non-invasive imaging tools can be used, including ultrasonography and magnetic resonance imaging.

Ultrasonography represents one of the cheapest, highly available, and non-invasive imaging tools for assessing endometriosis, either by transabdominal, transvaginal, or transrectal approach, being frequently used as an incipient screening test for endometriosis, as well as a preoperative tool for estimating the length of the surgical maneuvers [67]. This imaging technique is most used in evaluating the endometriotic cysts that usually present a varied sonographic spectrum ranging from anechoic cysts, cysts with diffuse low-level echoes to solid-appearing masses, that occasionally present septations, thickened walls, and wall nodularity, with pericystic blood flow [68]. Due to the high variation in sonographic characteristics, endometriosis lesions might be similar in appearance with other ailments such as dermoid cysts, hemorrhagic cysts, neoplasms, ovarian abscesses, and ectopic pregnancies, thus thorough differential diagnosis is needed to exclude the possibility of misdiagnosis [69].

To date, transvaginal ultrasonography represents the standard imaging technique for identifying ovarian endometriomas due to its high values in sensitivity (93%) and specificity (97%) when performed by an expert operator [70,71,72]. Nevertheless, transvaginal imaging has lower success rates in the visualization of adhesions or superficial peritoneal implants, thus a transabdominal approach or the use of magnetic resonance imaging can be more useful. Transrectal ultrasound has been also useful in aiding the endometriosis diagnostic, especially when assessing the extent of infiltration of the rectal and the posterior bladder walls [73]. 

Numerous attempts have been made to standardize the ultrasonographic method in order assure a complete evaluation of the lesions and in 2016, an international consensus group implemented a four-step systematic approach which includes four steps, respectively: in the first step a routine evaluation of uterus and adnexa is conducted, focusing on ultrasonographic features of adenomyosis and endometrioma; in the second step tenderness and ovarian mobility is evaluated; the third step involves assessment of the pouch of Douglas using the ‘sliding sign’; the fourth step focuses on exploring the anterior and posterior compartment, assessing deep infiltrative endometriosis nodules [74]. Despite all efforts, the main challenge of imaging endometriosis remains the detection of deep infiltrative lesions into the pelvic structures as well as non-ovarian cases, for which MRI evaluation is better suited [75].

Magnetic resonance imaging (MRI) is increasingly being used in the evaluation of patients with endometriosis, as a complementary method to transvaginal ultrasonography [76], particularly when the clinician questions the possible presence of deep infiltrative lesions. Other situations where MRI is useful, include the cases in which the sonographic features are elusive, or when surgery is programmed, as it provides a greater contrast resolution and a larger field of view compared to ultrasound [77]. The spectrum of endometriotic lesions which can be observed during an MRI examination is variated, some of the main lesion types being represented by superficial peritoneal implants, adhesions, endometrial ovarian cysts, and deep infiltrating endometriosis involving round ligaments, retro cervical region, the bladder or rectal wall [78]. The standard imaging protocols for acquiring images are the fast spin-echo sequences T2-weighted (T1W) and T1-weighted (T2W), helpful not only in detecting small lesions (lesser than 1 cm) but also in differentiating between the content of the cystic lesions when coupled with fat saturation and fat suppression protocols [79]. MRI characteristics of the endometriotic lesions consist either of high-intensity signal spots on T2W images that correlate to the endometriotic glands or high intensity-signal spots on T1W images, related to hemorrhagic foci in fibromuscular lesions [80].

Extensive efforts have been conducted to standardize the systems scores used to grade the disease’s extension by a consensus group focused on reproductive medicine that have materialized in the form of a classification system. This system assigns corresponding values to endometriotic lesions regarding the size of the lesions and the presence of adhesions material found on the gynecological structures. It is compartmentalized into four stages pertaining to the severity [81]. Despite its high sensitivity and specificity scores in detecting endometriosis, MRI presents some limitations in regard to its performance, mainly in cases of intestinal deep infiltrating endometriosis by reduced bowel peristalsis, in cases of anatomical variations of structures such as the rectovaginal septum or in the situation of a retroflexed uterus. Such events may impair the accuracy of diagnostics, thus calling for a surgical approach in aiding the assessment of the disease extent [82].

Surgical exploration represents an invasive visualization technique used specially to acquire qualitative assessment data about the true extent of the endometriotic lesions. Despite all available imaging techniques, the laparoscopic inspection along with histologic confirmation [83], remains the gold standard for confirmatory diagnosis of endometriosis. Laparoscopy enables the direct visualization not only of the more superficial implants and endometriomas but also of the adhesion pattern as well as deep infiltrative lesions of the bowel or the urinary system [84]. The classic appearance of the peritoneal implant is of a blue-black “powder burn” or “shotgun” lesion (resulting from hemosiderin deposits due to entrapped menstrual debris), red or white lesion, while endometriomas can present as smooth-walled, dark, brownish cysts, both of the lesions being strongly associated with the presence of peri adnexal adhesions [55,85,86]. The extent of deep infiltrating endometriosis is difficult to assess during diagnostic laparoscopy alone and can be greatly appreciated during operative laparoscopy.

During the last decades, enhanced laparoscopic imaging techniques have emerged, showing improvement in the detection and differentiation of specific tissues with the aid of either of narrow-band imaging (NBI), autofluorescence (AFI), or 5-aminolevulinic acid-induced fluorescence (5-ALA) [87]. Even though diagnostic laparoscopy represents the standard procedure to date for confirmation of endometriosis diagnostic, it still carries an estimated risk of death at 0.1/1000, due to the possibility of injuring abdominal structures such as the intestine, ureter, or the major blood vessels. In this case, conversion to laparotomy is required, adding up unfortunately to the medical burden of the patient as well as the total costs of the procedure [88].

## 3. Mechanisms Involved in the Pathogenesis of Endometriosis-Associated Infertility

Understanding the intricate mechanisms concerning the etiopathogenesis of endometriosis and infertility as a complication, was an enigmatic task in the era of limited diagnostic techniques, when the genetic, cytogenetic, and molecular tools were not yet fully developed. Although several studies have been focusing on the topic, there still little agreement regarding all the aspects that link these two medical entities. To date, in the case of not objectifying anatomical distortions such as adhesions and fibrotic tissue, few mechanisms shed light on the exact steps that lead to infertility [89].

**Figure 1:**
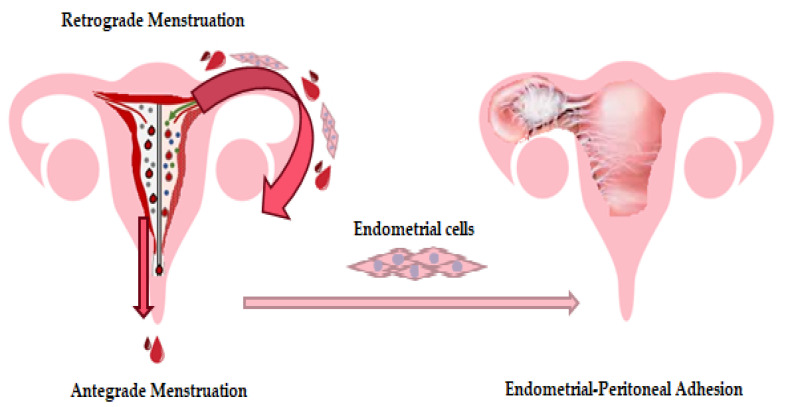
As the theory of retrograde menstruation stipulates, the implantation of viable endometrial cells in the peritoneal cavity is facilitated by the constant reflux of endometrial debris during menstruation, leading to an inflammatory microenvironment in this anatomical compartment.

Over the years, the most acknowledged theory concerning the etiopathogenesis of endometriosis was that of the retrograde menstruation, described by the endometrial cell implantation in different peritoneal locations, cells that sidestep the fallopian tubes and implant in the peritoneum, where the newly created immune microenvironment aids in the survival of these cells (Figure 1) [90].

While endometriosis is a chronic inflammatory disease that involves the presence of extrauterine endometrial-like tissue, adenomyosis is a benign condition that occurs at the level of uterus, within the myometrium, being characterized by the presence of ectopic endometrial tissue glands and stroma in this area [91,92]. This particular condition is usually diagnosed while investigating with other gynecological comorbidities such uterine fibroids and endometriosis [92,93,94]. Several hypotheses have been proposed, but a common definition of adenomyosis remains controversial due to theories regarding the pathogenesis, diagnostic criteria, and classifications of this disease. 

Although adenomyosis has many features in common with endometriosis and often coexists [95,96,97], they are considered two different pathologic entities. Following numerous studies on these two gynecological diseases so similar that adenomyosis was called internal endometriosis [97,98], many differences in clinical manifestations, risk factors, and pathogenesis were discovered. However, there are many similarities between the two conditions in terms of molecular aberrations, hormonal and immune system disorders, and clinical manifestations. 

Regarding female fertility, it has been suggested that 30–50% of those diagnosed with endometriosis are infertile [99], with an increase of up to 80% in such patients following assisted reproduction techniques (ARTs) [100]. Although adenomyosis has been considered a typical uterine condition identified in multiparous women over the age of 40, recent studies and modern diagnostic imaging methods have demonstrated the presence of this disease in young women as well. Regarding the association with infertility and reproductive failure the evidence suggests that the percentage of adenomyosis prevalence is variable between 20% and 40% in cases of recurrent pregnancy loss, and about 35% was reported in previous ART failure [101,102,103,104,105].

Functional and structural changes in the eutopic endometrium and inner myometrium are characteristic of endometriosis and adenomyosis, these changes having negative consequences for female fertility. Altered endometrial receptivity in patients with endometriosis and adenomyosis is also linked to some molecular events that are associated with the process of implantation and embryo development [91].

Disorder of these processes is associated with an increased likelihood of abnormal endometrial molecular expressions of genes that are part of the homeobox (Hox) gene family, as well as other autocrine and paracrine factors, growth and transcription factors, steroid hormones, molecules cell adhesion, immune and inflammatory mediators, and other factors, including myometrial contractility and uterine peristalsis [91,102].

Along with the technological advancements, several new mechanisms that could explain this link have been proposed such as endocrine and immunological abnormalities. From an endocrine perspective, most of the theories take into consideration defects in folliculogenesis and ovulation, as well as abnormal hormonal serum levels such as hyperprolactinemia. Mechanisms concerning the immunological alterations focus on events like sperm phagocytosis, embryotoxicity along with implantation defects that happen due to alteration on a molecular level [106]. Nevertheless, several factors complicate the advancements of this research domain due to matters related to the disease such as the phenotypic heterogeneity and the higher underdiagnostic rates compared to other diseases as well as management issues due to the lack of thorough indexing and registering on a national and international scale.

### 3.1. Genetic and Molecular Mechanisms Concerning the Pathogenesis of Endometriosis

Even though the theory of retrograde menstruation [90] is still accepted as the most feasible explanation for the occurrence of endometriosis, evidence sustains newer theories such as the “embryonic theory” or epigenetic theory. This hypothesis takes into consideration that the during organogenesis, some of the genes involved in the differentiation of the urogenital tract might become dysregulated, leading thus to anatomical abnormalities, as well as misplacements of the stem cells [107].

The first studies regarding the genetic predisposition of this ailment were conducted in 1981 when Simpson and colleagues [108] observed that 61% of the women enrolled in the study had a first-degree relative (mother or sister) suffering from severe endometriosis and only 23% of the women with endometriosis did not have such connections [30,109,110]. Despite numerous discrepancies concerning the prevalence of endometriosis in first-degree relatives cited in different publications, several other studies demonstrated the existence of a hereditary pattern [111,112,113,114,115,116,117,118,119]. It has been confirmed that the intricate gene interactions concerning not only the regulation of the cell cycle but also of the synthesis of various molecules such as growth factors, hormone receptors, and adhesion molecules, constitute a special genetic status that predisposes women to develop endometriosis [71,113,120]. Cohort studies of population sets from various regions around the world, showed that some genotypes are correlated with increased endometriosis prevalence. After analyzing various population groups from countries such as of Sardinia, Turkey, Brazil, Taiwan, or Australia, it has been suggested that the cause of higher prevalence of endometriosis might rely on interactions between specific risk alleles [110,117,120,121].

Recent findings of Rekker et al. (2018), suggested that changing the miRNA expression patterns of the endometrial stromal cells could to be involved in the pathogenesis of endometriosis [122]. In their endeavors, the research team discovered 21 miRNAs with such modified expressions, one of them being miR-139-5p. This miRNA has a direct implication in regulating the homeobox gene, presenting a suppressive outcome on both the HOXA9 and HOXA10 genes [122,123]. Boergese et al. and several other researchers published studies in regard to miRNA and its expression in the lesions of endometriosis, with the summarized results being shown in Table 1. Even though the role of microRNAs has been intensely investigated, there is still inconsistent data regarding the regulation of processes involved in generating the endometriotic lesions, since the cells exhibit heterogeneous miRNA signatures with various properties [44].

Many studies have been performed to identify the polymorphism of genes involved in various molecular pathways specific to the pathogenesis of several diseases, especially those with an inappropriate immunological response, such as endometriosis.

Among the pathways involved in the immune response that are disrupted in autoimmune diseases, inflammatory processes, allergies, and endometriosis, the family of genes that regulate Toll-like receptors (TLRs) [133,134], types of molecules which regulate the activation of immune response and inflammation pathways that could be also dysregulated. To date, more than 13 members of TLRs family have been identified, the most studied one being Toll-like receptor 4 (TLR4) and Toll-like receptor 2 (TLR2) [134,135,136], although the presence of TLR 1 to TLR 6 (TLR1, 2, 3, 4, 5, and 6), was reported in epithelia of different regions of endocervix, endometrium, and uterine tubes in patients with endometriosis [135,137,138].

TLR receptors regulate the activity of immune and inflammatory factors that modulate the innate immune response, and the activation of the persistent inflammation phase by triggering oxidative stress. Due to activation of TLR4 signaling pathway, some specific process for endometrial proliferation are initiated by secretion of cytokine and other specific immune cells responsible for the regulation of inflammatory microenvironment [139]. Some studies have shown that TLR4 expression is decreased in patients with endometriosis [135,136,137,138,140].

### 3.2. Biochemical and Immunological Traits that Associate Infertility to Endometriosis

The link between infertility and endometriosis is complex, and understanding it implies unraveling multiple intricate pathways. From a biochemical perspective, oxidative stress represents one of the most researched factors linked to endometrial induced infertility. Aberrant increased levels of reactive oxygen species (ROS) resulted from oxygen metabolism impact the endometrial cells by damaging not only proteins and lipids but also DNA structure, thus altering the cellular cycle and function. One study focused on assessing the biochemical imbalances for women undergoing surgery for gynecological problems, found increased serum levels of such markers including advanced oxidation protein products as well as nitrates/nitrites, especially in patients suffering from advanced stages of endometrial disease [107].

As with any other chronic inflammatory affliction, endometriosis has been proved by several studies to be characterized by a complex landscape of increased levels of inflammatory molecules such as cytokines, prostaglandins, and metalloproteinases, aiding thus in the development and progress of the disease [141,142,143]. Some cytokines and chemokines found in increased levels in the serum of patients suffering from endometriosis are thought to participate in the generation of the inflammatory status, of which interleukin-1β, interleukin-6, tumor necrosis factor (TNF), monocyte chemoattractant protein 1, and RANTES, represent some of the most studied molecules [144,145]. As a final step to any inflammatory process, fibrosis, a main characteristic of endometriosis, is heavily influenced by the interplay of the inflammatory molecules. From generating the granulation tissue to myofibroblastic differentiation, several molecules have been identified to take part in the disease’s progression, of which alpha-smooth muscle actin (α-SMA) represents a highly studied marker [146,147]. Although much interest has been focused on this matter because of its clinical relevance, the mechanisms by which fibrosis is initiated are still unclear. Several studies on the inflammatory environment brought also into light that several molecules such as adhesion molecules, VCAM-1 and ICAM-1, and metalloproteinases, MMP-2, MMP-3, MMP-7, and MMP-9, presented increased levels in the serum of patients suffering from endometriosis [148,149,150].

Regarding the theory of immunological dysfunction, several research studies focused on the role of humoral and cellular immunity in the generation and progression of the disease [150,151], found that aberrant responses are present throughout a wide range of cells including not only neutrophils, macrophages, and natural killer cells behavior, but also T cells and B cells [152]. It is believed that ectopic endometrial cells refluxed in the peritoneal compartment during the retrograde menstruation cannot induce immunologic activation that leads to their destruction [153]. One study investigating the implications of neutrophils found that lower apoptosis rate was one of the characteristics of these cells in the endometriotic tissues, suggesting the presence of antiapoptotic factors in the plasma of women suffering from the disease [154]. Macrophages have been the focus of several studies that demonstrated progression of the disease by stimulating angiogenesis through increased production of VEGF, IL-8, and TNF alpha [155,156]. Recent studies also reported that IL-6 might alter NK cells cytotoxicity, thus contributing to the immunologic dysfunction [157]. Several other cytokines and chemokines have been shown to be present in increased serum levels in patients suffering from endometriosis, of which some of the most researched molecules are CCL5, CCL2, CXCL1, CXCL8, CXCL5, CXCL12 and IL-1, IL-6, IL-8, IL-12 [158]. In relation to the cell-mediated immunologic alterations, studies have focused especially on the role of T helper cells, cytotoxic T cells, and B cells. Numerous research papers documented that cytotoxic T cells present a low proliferative response, while T helper cells show increased activity in regard to the ectopic endometrial cells [159,160,161]. B-cell activation showed to be abnormal, suggested not only by the increased levels of IgG and IgA, but also by the increased production of several antibodies such as anti-nuclear antibodies, anti-DNA antibodies, and anti-phospholipid antibodies, suggesting this way that endometriosis might as well be an autoimmune disease [162].

## 4. Therapeutic Options Available for Endometriosis-Associated Infertility

There are three therapeutic options available as a therapy for endometriosis-associated infertility: medical treatment [163], surgery [164], and technologies adapted for assisted reproduction [165].

### 4.1. Medical Treatment Options

Medical therapy for patients with endometriosis-associated infertility involves two strategies, with the main purpose of improving fertility: either stimulation of ovulation and of the follicular development process or suppression of follicular development to generate amenorrhea and inhibit the enlargement of endometriotic lesions. A Cochrane review showed that suppressing ovulation using gonadotropin-releasing hormone (GnRH) agonists, oral contraceptives, progestins, and danazol is not considered a suitable therapeutic option for women suffering from infertility associated with endometriosis due to similar outcome regarding pregnancy when compared with women on placebo or no treatment [166].

Medical treatment with these therapeutic agents tends to ameliorate pain symptoms, but they usually cause subfertility and therefore are not useful for patients with endometriosis-associated infertility with the purpose of increasing pregnancy rates and live births [99,167].

For ovulation induction, clomiphene citrate has been the most widely prescribed treatment, either alone or combined with gonadotropins [99,167]. Another medical treatment for endometriosis includes aromatase inhibitors, that also stimulate the ovary, but they cause functional ovarian cysts, so in premenopausal women, they should be administered in combination with GnRH agonist, progestins, or combined oral contraceptive [168,169].

Studies have shown that prolonged treatment with GnRH agonists before IVF or ICSI may improve pregnancy outcomes in women suffering from advanced forms of endometriosis. Pre-treatment with GnRH agonists can improve the oocyte quality and the ovarian microenvironment [7,170,171].

Choosing the best suited therapeutic option for endometriosis-associated infertility represents a challenging task since various parameters such as the woman’s age, ovarian reserve, duration of infertility, the presence of pelvic pain, family history, ability to undergo IVF have not been properly evaluated in randomized clinical trials [10,172].

For younger infertile women (under 35 years) with minimal-mild endometriosis, a good first-line treatment is represented by expectant management or the use of supraovulation (SO) with intrauterine insemination (IUI). Older women with stage I–II endometriosis-associated infertility can greatly benefit from be a more aggressive treatment with SO/IUI or IVF. For infertile patients suffering from stage III–IV endometriosis, conservative surgery with laparoscopy or possible laparotomy could be the best available therapeutic option. IVF may be an efficient treatment for women with moderate to severe endometriosis, if all the aforementioned treatments do not meet the desirable fertility outcome [172].

It was proven that prolonged treatment with GnRH agonists before IVF or ICSI may improve pregnancy outcomes in women suffering from advanced forms of endometriosis. Pre-treatment with GnRH agonists can improve the oocyte quality and the ovarian microenvironment [7,170,171].

### 4.2. Surgical Treatment Alternatives

Surgical treatment options in endometriosis-associated infertility are laparotomy, laparoscopy, or robotic surgery. Laparoscopic intervention is most frequently used due to its advantages, including a lower cost and a shorter recovery and hospitalization [7]. Surgical intervention aims to remove endometriotic implants and endometriomas and restore the normal pelvic anatomy to the greatest possible extent [173]. Literature data have shown that laparoscopy surgery in minimal-mild endometriosis improves fertility and live birth rates [174,175]. In moderate-severe endometriosis, the laparoscopic surgery can treat pelvic adhesions, but unfortunately, there are insufficient randomized controlled trials on postoperative pregnancy rate [100,176].

Studies have shown that surgical treatments have a series of potential disadvantages, such as surgical complications, a decline in ovarian reserve, postoperative adhesions, a possible postponement in infertility treatment [100,177]. For ovarian endometriomas greater than 3–4 cm, excisional surgery has shown better outcomes than ablation and drainage regarding spontaneous pregnancy rates in women with previous subfertility. Furthermore, excisional surgery was correlated with a decreased rate of recurrence and better pain relief [178,179]. A new technique concerning the treatment of endometriotic lesions is robot-assisted laparoscopy. Despite higher costs, statistics did not show it provides more benefits than standard laparoscopy [180].

Newer studies suggest that laparoscopic surgery as an investigative and corrective tool of the underlying pathologies prior to initiating numerous and various therapeutic attempts, should be taken into consideration for women suffering from unidentified infertility etiology, as it can greatly impact the outcome, as well as improving the overall costs of the lengthy treatment plan and ultimately the quality of life of the women going through this journey towards child conceiving [181]. The same view on laparoscopic investigation prior to concentrating on assisted reproduction techniques is shared when focusing on mild male factor infertility cases, especially if previous failed IVF have attempts attributed to an infertility etiology [182].

### 4.3. Assisted Reproductive Technology (ART)

ART includes a number of treatment methods that combine follicle stimulation with the handling and preparation of gametes in order to overcome infertility-related problems. This technology is comprised of in vivo or in vitro methods. In vitro fertilization implies that the oocytes are extracted, that they are fecundated and grown in the laboratory before being transferred back into the uterus. The most common in vivo technique is intrauterine insemination (IUI) that includes follicle stimulation or not, followed by the transfer of the semen into the uterine cavity. The most frequently in vitro method used in couples with a standard sperm count is in vitro fertilization (IVF). In situations of severely decreased sperm quality, an intracytoplasmic sperm injection can be used as a therapeutic option to increase rates of a good fertility outcome (ICSI) [183].

#### 4.3.1. Intrauterine Insemination

IUI is a relatively simple method and has been subject to a number of studies regarding couples characterized by milder stages of endometriosis and normal semen quality. Since tubal pathology is one of the most frequent causes of infertility, it is essential to assess the tubal patency prior to IUI procedure. Hysterosalpingograpy (HSG) is commonly used as the first-line of action to evaluate the patency of the fallopian tubes and the uterine anatomy [184,185].

Patients with minimal to mild endometriosis that have been surgically diagnosed and have not shown any anatomic distortion, were directed to ovarian stimulation (with gonadotropins or clomiphene citrate) followed by IUI, which can be a suitable alternative to IUI alone or IVF [100,186,187].

Women suffering from moderate to severe forms of endometriosis do not benefit from IUI, on account of a probable impact on the uterine tubes. Consequently, it can be more relevant to compare minimally to mild endometriosis-associated infertility with unexplained infertility. Infertile patients suffering from mild forms of endometriosis achieve lower pregnancy rates following controlled stimulation and IUI than patients with unexplained infertility [188]. Nevertheless, after the surgical intervention in cases of women suffering from stage I and II endometriosis, the pregnancy rate per therapy cycle and the cumulative live-birth rate were comparable in endometriosis and idiopathic infertility patients, indicating that endometriosis affects fertility [189].

Studies have showed that for every 12 women with minimal to mild endometriosis-associated infertility that underwent laparoscopic interventions for ablation of the endometrial implants, an additional pregnancy might develop when compared with the situation of no administered treatment. The number of laparoscopic interventions required to obtain an additional pregnancy in asymptomatic women with unexplained infertility is estimated around 40 [172,190].

#### 4.3.2. In Vitro Fertilization

Currently, IVF is the most successful treatment for infertile women with endometriosis and includes a few steps. The first step is ovarian stimulation initiated by drugs and the suppression of the menstrual cycle using other medications. After follicle stimulation, monitoring is performed at certain intervals to evaluate the follicle’s growth. Once the follicles have reached a proper dimension, medication is administered to induce the final stage of maturation of the oocyte. The next step implies collecting the egg, followed by the fertilization process. In cases of male infertility, the fertilization is completed by ICSI. The fertilized egg is cultured in a specific media for a few days and then the embryos are transferred into the uterus [191].

A meta-analysis has shown that patients suffering from endometrioma that underwent IVF had comparable live birth rates as well as clinical pregnancy and miscarriage rates when compared to women that were not affected by this disease, even though the mean number of oocytes and the antral follicle count were lower and the risk of cycle cancellation was increased in women with endometrioma [192]. Studies reported that there is no difference regarding IVF results between patients with reduced ovarian reserve after ovarian surgery and those with reduced ovarian reserve without prior endometrioma surgical treatment [160]. Whereas the live birth rates do not seem to be influenced by surgery, surgical intervention of endometrioma previous to IVF/ICSI may cause a more detrimental effect on ovarian reserve [10,193].

A 2020 meta-analysis investigated the efficiency of the GnRH-a short protocol, GnRH-a long protocol, and GnRH-a ultra-long protocol used in IVF-embryo transfer in women suffering from endometriosis-associated infertility. The analysis of the randomized controlled trials has revealed that the clinical pregnancy outcome, especially in women with moderate to severe forms of endometriosis was significantly higher in GnRH-a ultra-long protocol group when compared with the GnRH-a long protocol group. On the other hand, in the observational studies no significant difference was observed between the two groups regarding the pregnancy rate [144].

Systematic reviews reported that second-line conservative surgery for recurrent endometriosis has a negative effect on the IVF outcomes. The number of mature oocytes retrieved following IVF procedure and high-quality embryos was considerably reduced after second-line conservative surgery than after the initial conservative surgical intervention. Second-line surgery seems to significantly diminish the ovarian reserve and therefore this procedure should be attentively considered as an alternative therapeutic method of women who desire a future pregnancy [194,195].

## 5. Future Perspectives

The majority of medical treatment options available for endometriosis are suppressive rather than curative and symptoms reappear when medication is interrupted, therefore there is a need for novel developments in this domain. Elagolix, an oral anti-gonadotrophic agent is a novel and promising therapeutic method for endometriosis, that seems to stop the evolution of the disease and significantly reduce pain [196]. Resveratrol is a natural compound that possesses anti-angiogenic, anti-carcinogenic, proapoptotic, anti-oxidative, and anti-inflammatory effects and might provide novel therapeutic perspectives in endometriosis treatment [197]. Studies suggested that antioxidants such as melatonin, vitamins E and C may also be helpful to current endometriosis therapies [198].

Therapy based on mesenchymal stem cells has been applied in infertility treatment for women with premature ovarian failure and Asherman’s syndrome [199]. Due to its immunomodulatory and tropic effects against inflamed lesion foci, stem cell therapy is an attractive therapeutic option for endometriosis. Stem cell therapy is a prospective option to replace injured endometrium. However, this treatment had caused controversy concerning the stem cell implication in the disease’s pathogenesis [200].

Multiple studies have indicated that circulating miRNAs are promising candidates as non-invasive biomarkers in the diagnosis of endometriosis [201].

## 6. Conclusions

Endometriosis is a disorder that affects women of reproductive age, causing pain and infertility problems. Even though the association between infertility and endometriosis is still controversial, it is clinically recognized and well supported by many studies.

The treatment of endometriosis-associated infertility consists of reducing or removing the ectopic endometrial implant and restoring normal pelvic anatomy through medical and/or surgical treatment and assisted reproduction technology. Medical treatments of endometriosis-associated infertility tend to ameliorate pain symptoms, but they are not effective in infertility treatment. These treatments should be utilized as an adjuvant to ART. 

ART includes IUI and IVF and comes into play when neither medical nor surgical therapy meets the desired outcome. IUI is efficient in patients with minimal to mild endometriosis, that have been surgically diagnosed and have not shown any anatomic distortion. IVF has shown to be the most successful treatment for infertile women with severe endometriosis.

## Figures and Tables

**Table 1:** Pathophysiological processes altered by miRNA molecules, correlated with the modified expression in endometriosis suffering patients.

Pathophysiological Processes Altered by miRNAs	Downregulated	Upregulated	Published Studies
Cellular proliferation and differentiation Apoptosis Angiogenesis Matrix remodeling	miR-543, miR-20a, miR-34c, miR-221, miR-222	miR-142-5p, miR-146-a-5p, miR-1218, miR-940, miR-4634, miR-125a, miR-125b, miR-126, miR-143, miR-145	[124,125,126,127]
Angiogenesis Extracellular matrix remodeling Tissue repair	hsa-miR-483-5p, hsa-miR-483-5p, miR-16, miR-20a, miR-21, miR-141	miR-202-3p, miR-424-5p, miR-556-3p, miR-449b-3p, miR-449b-3p, miR-556-3p, miR-29c-3phsa-miR-24, hsa-miR-885hsa-miR-26b, hsalet-7b, hsa-miR-18, miR-1, miR-194, miR-29c	[124,125,128]
Hypoxia Apoptosis	miR-15b, miR-16, miR-199a, miR-20a, miR-200, miR-424, miR-130	miR-20a, miR-20b, miR-155	[124,129,130,131]
Targeting inflammation	miR-16, miR-20a, miR-199a	miR-302a, miR200a	[124,130,131,132]
